# Genetic Mapping and Identification of the Candidate Genes for Mottled Rind in *Cucumis melo* L.

**DOI:** 10.3389/fpls.2021.769989

**Published:** 2021-11-15

**Authors:** Jia Shen, Xinyang Xu, Yuejian Zhang, Xiaowei Niu, Weisong Shou

**Affiliations:** Institute of Vegetables, Zhejiang Academy of Agricultural Sciences, Hangzhou, China

**Keywords:** melon, mottled rind, spots, chlorophyll, mapping

## Abstract

The rind appearance of melon is one of the most vital commercial quality traits which determines the preferences and behavior of consumers toward the consumption of melon. In this study, we constructed an F_2_ population derived from SC (mottled rind) and MG (non-mottled rind) lines for mapping the mottled rind gene(s) in melon. Genetic analysis showed that there were two dominant genes (*CmMt1* and *CmMt2*) with evidence of epistasis controlling the mottled rind. Meanwhile, the phenotypic segregation ratio implied that the immature rind color had an epistatic effect on the mottled rind, which was regulated by *CmAPRR2*. A Kompetitive Allele-Specific PCR (KASP) DNA marker (*CmAPRR2*^*SNP(G/T)*^) was developed and shown to co-segregate with rind color, confirming that *CmAPRR2* was *CmMt1*. Using bulked segregant analysis sequencing and KASP assays, *CmMt2* was fine-mapped to an interval of 40.6 kb with six predicted genes. Functional annotation, expression analysis, and sequence variation analyses confirmed that *AtCPSFL1* homolog, *MELO3C026282*, was the most likely candidate gene for *CmMt2*. Moreover, pigment content measurement and transmission electron microscopy analysis demonstrated that *CmMt2* might participate in the development of chloroplast, which, in turn, decreases the accumulation of chlorophyll. These results provide insight into the molecular mechanism underlying rind appearance and reveal valuable information for marker-assisted selection breeding in melon.

## Introduction

Melon (*Cucumis melo* L. 2*n* = 24) is an economically important vegetable crop grown throughout the world. Based on ovary pubescence, melon has been classified into two subspecies, *C. melo* ssp. *agrestis* and *C. melo* ssp. *melo* (Jeffrey, 1980). Recent studies revealed that there are two independent sets of domestication sweeps, resulting in diverse characteristics of these two subspecies during melon cultivation, especially the fruit characters (Zhao et al., 2019; Liu S. et al., 2020). External fruit (rind) appearance is an important commercial quality trait affecting the consumption of melon of consumers, which has abundant genetic diversity (Pitrat, 2016). In recent years, numerous genetic studies have focused on the rind appearance of melon, including rind color and rind pattern (Feder et al., 2015; Pereira et al., 2018; Oren et al., 2019; Zhao et al., 2019).

For convenience, the rind color could be divided into immature fruit and mature fruit color according to the development stage (Pitrat, 2016). Previous studies have shown that besides chlorophyll, the pigment regulation of immature rind color is inhibited early in fruit development, reflecting chlorophyll concentrations (Tadmor et al., 2010), and that rind color transforms during development leading to rich variation in mature fruit pigment profiles that include different combinations of carotenoids, flavonoids, and chlorophylls (Tadmor et al., 2010; Feder et al., 2015). Therefore, compared with mature fruits, the rind color of immature melon fruits is relatively straightforward, dividing into light green and dark green (Pitrat, 2016). And the causative regulatory gene (*CmAPRR2*) of chlorophyll accumulation in the immature fruit rind of melon was identified (Oren et al., 2019). Xu et al. (2021) cloned the same gene with the genetic population constructed between subspecies, indicating the conservation of the *CmAPRR2*.

As for rind pattern, the presence of spots on the rind is an important rind pattern trait commonly found in commercial melon (Pitrat, 2016). Although the study of the genetic pattern of spots on the rind by multiple research groups is relatively early (Danin-Poleg et al., 2002; Zhao et al., 2019), they have given unconvincing and even conflicting results. A single gene, *Mt* (Mottled rind pattern), was reported to control the mottled rind, dominant to uniform color *mt* (Ganesan, 1988), while Perin et al. (2002) showed that the presence of dark spot on the rind has a monogenic recessive inheritance (*mt-2*). And *mt-2* was mapped to the linkage group II (Pitrat, 2008). Since the reference genome of melon was released in 2012 (Garcia-Mas et al., 2012), we were unable to define the physical position on the reference genome. And then, Pereira et al. (2018) detected that the mottled rind was controlled by a dominant gene *Mt*, which was identified on chromosome 2 within 850 kb by a genotyping-by-sequencing strategy. Recently, Lv et al. (2018) narrowed the *Mt* positioning interval to 280.872 kb of chromosome 2, although the interval was not completely coincident. Interestingly, it was also mapped on chromosome 4 within 172.8 kb (Liu et al., 2019). Thus, the mottled rind of melon may be relatively complex, and more effort should be required for the fine mapping and cloning of the *Mt* gene(s).

Research on melon rind appearance is very important both in theoretical studies and in applied practice for elucidating the mechanism of mottled rind and for the breeding of melon. In this study, two inbred accessions, namely, *C. melo* ssp. *agrestis* (SC, mottled rind) and *C. melo* ssp. *melo* (MG, non-mottled rind), were used to identify the pigment components of the dark spots formation on the melon rind. The inheritance of mottled rind was analyzed, and two dominant genes (*CmMt1* and *CmMt2*) with evidence of epistasis were responsible for the mottled rind. *CmAPRR2* was the candidate epistatic gene for *CmMt1*, and a candidate gene encoding *AtCPSFL1* homolog, *MELO3C026282*, was predicted by map-based cloning for *CmMt2*. The present results may highlight the understanding of the genetic basis for mottled rind formation and promote the breeding of melon with ideal rind appearance by marker-assisted selection.

## Materials and Methods

### Plant Materials and Phenotypic Data Collection

An inbred line of *C. melo* spp. *agrestis* (Songwhan Charmi, SC) and an inbred line of *C. melo* ssp. *melo* (Mi Gua, MG) were employed to generate F_1_, BC_1_, and F_2_ populations in this study. The female parent was mottled and dark green rind, while the male parent was uniform and light green rind. The SC and MG lines as well as their F_1_, F_2_, and BC_1_ populations were used for genetic analyses. The F_2__:__3_ families were developed and used in this study for the fine mapping. All the plants were hand pollinated with two staminate flowers and clamped with grafting clips to prevent re-pollination by entomophily or pollen dispersal. All the lines were grown in the plastics greenhouse at Zhejiang Academy of Agricultural Sciences under the natural sunlight in spring 2019 in Yangdu (120°46′ E, 30°45′ N).

Each plant, including the parental lines as well as F_1_, BC_1_, and F_2_ individuals, was given an identification tag revealing its serial number, pollination date, and cross-combination type. The phenotype of the fruit rind was detected through visual observation and a digital portable colorimeter (Minolta CS-321) at 10–15 days after pollination (dap). And each fruit was photographed for reassessment. The young leaves of each plant were cut from the plant apex and flash-frozen in liquid nitrogen and maintained at −80°C for further use.

### Pigment Content Measurement and Transmission Electron Microscopy

According to the initial stage of phenotypic differences between parents, rind samples from the immature fruits of melon at 15 days after pollination were collected to measure the chlorophyll and carotenoid contents. The pigment content was measured on three biological replicates for each sample analysis, each sample comprising three individual fruits.

The same samples were prepared for transmission electron microscopy (TEM). The samples were vacuumed in 2.5% glutaraldehyde, fixed at 4°C for 4 h, and then dehydrated in a graded ethanol series, and critical point drying was performed using liquid CO_2_ in a Bal-Tec CPD 030 critical point drier. Later, the samples were coated with 15 nm gold on aluminum stubs in a Sputter Coater Bal-Tec SCD 005. A Hitachi S-3500N scanning electron microscope was subsequently used for sample observations. The chloroplast number and size in the rind cells (*n* = 10 for each sample) was measured by ultrathin section with CaseViewer 2.1 (3DHISTECH, Budapest, Hungary).

### Bulked Segregant Analysis Sequencing

Bulked segregant analysis sequencing (BSA-seq) was used to map the *CmMt* gene. The genomic DNA was extracted from young leaves of F_2_ individuals using the CTAB (Murray and Thompson, 1980). A mottled pool containing equal amounts of DNA from 20 mottled rind individuals and a uniform pool containing 20 individuals with uniform rind were prepared for whole-genome resequencing with an Illumina HiSeq 2500 (500 bp) in Novogene (Beijing, China). The clean data obtained from the DNA pools were aligned with the “DHL92” melon reference genome (v4.0) (Castanera et al., 2019). Variant calling including single-nucleotide polymorphism (SNP) and insertion and deletion polymorphisms (Indels), SNP and Indel filtering, index calculation, and mapping region identification were performed as described by Klein et al. (2018).

### Fine Mapping With Kompetitive Allele-Specific PCR Markers

According to the BSA-seq analysis, 25 SNPs in the candidate region were selected for developing Kompetitive Allele-Specific PCR (KASP) marker. The KASP primers were validated with the two-parent lines and a small number of F_2_ individuals first. Then, a total of 553 F_2_ individuals with two parents were genotyped by the KASP markers, and the primers are shown in Supplementary Table 1. The linkage map was constructed using QTL IciMapping 4.2 (CAAS, Beijing, China) (Meng et al., 2015). The KASP assays were performed as described previously (Yang et al., 2020). Briefly, KASP reaction mixture contained 2.0 μl of template DNA, 0.07 μl of primer mixture (10 μM), 0.43 μl of RNase-free water, and 5 μl of KASP master mixture (2×; LGC, Middlesex, United Kingdom). The PCR cycling started with an initial denaturation at 95°C for 15 min, followed by 10 cycles for 20 s at 95°C, 60 s at initially 65°C, decreasing by 1°C per cycle, finally by 30 cycles for 10 s at 95°C and 60 s at 57°C. The components required for the KASP reaction were added to a 1,536-well plate. Two fluorescence, namely, FAM and HEX, were selected for the PCR reaction in the CFX Connect^TM^ Real-Time System and the terminal fluorescence signal was read after the reaction.

### Real-Time Quantitative PCR Analysis

The rind of the parents had been collected for real-time quantitative PCR (RT-qPCR) from the immature fruits of melon at 15 days after pollination, when there were phenotypic differences between parents. For each parent, the rind samples from three individual plants were used as one biological replicate, with a total of three biological replicates. The RT-qPCR was performed with the StepOne PCR System (Thermo Fisher Scientific, Waltham, MA, United States), and amplification was conducted using Maxima SYBR Green qPCR Master Mix (Thermo Fisher Scientific, Waltham, MA, United States) with 40 cycles of denaturation at 94°C for 30 s, annealing at 60°C for 30 s, and extension at 72°C for 30 s. The amplification specificity was tested by a dissociation curve (65–90°C) to compare the results from different reactions and samples. The primers are shown in Supplementary Table 2. The expression levels of genes were normalized to the value for *Actin* (*MELO3C008032*). At least three independent biological and technical replicates were tested per sample. The primer efficiencies were estimated with a dilution series, and the relative gene expression levels were determined by 2^–ΔΔCt^ formula (Livak and Schmittgen, 2001).

### Statistical Analysis

The mean values and SD of pigment contents and the expression levels of the related genes were calculated based on three independent biological replicates. Significant differences (*p* < 0.05) were statistically determined by Student’s *t*-test and Duncan’s method performed on SPSS version 20.0 software (IBM, Chicago, IL, United States).

## Results

### Phenotypic Characterization of the Melon Rind

**FIGURE 1:**
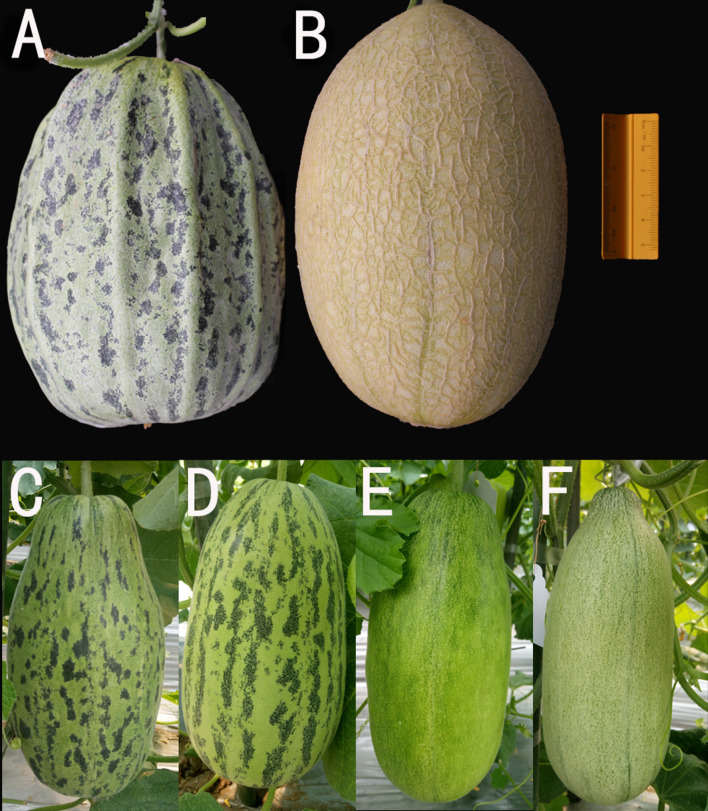
Mature melon fruits of the parents and immature melon fruits of F_1_ and F_2_ individuals. **(A)** SC, the maternal parent with mottled rind (35 dap). **(B)** MG, the paternal parent with non-mottled rind (40 dap). **(C)** F_1_ hybrid from SC × MG. **(D–F)** F_2_ individuals with the typical phenotype (15 dap).

The phenotypic analyses showed significant differences in rind pattern between the two parental lines. Compared with the light green and non-mottled (uniform) rind of MG (P_2_, Figure 1A), SC (P_1_, Figure 1B) showed mottled with spots on the dark green rind at the immature fruit stage (visible approximately 10–15 dap). The spots were distributed longitudinally and stable even after fruit ripening.

**FIGURE 2:**
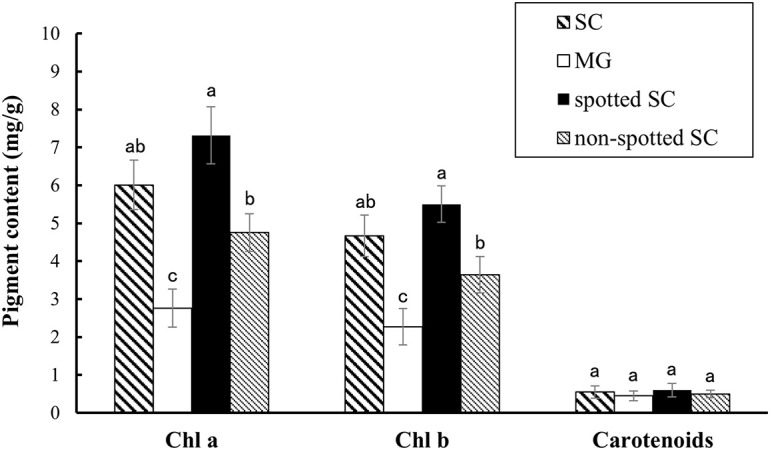
Pigment contents analysis. Contents of chlorophyll and carotenoids in the 15-dap peels of two parental lines, SC and MG. All data are shown as mean ± SD of three replicates. Different letters indicate significant difference (Duncan’s multiple range test, *p* < 0.05). Spotted SC, spotted part of SC peel; non-spotted SC, non-spotted part of SC peel; Chl *a*, chlorophyll *a*; Chl *b*, chlorophyll *b*.

Pigment content measurement showed that the rind of SC had a higher chlorophyll content, including Chl *a* and Chl *b*, than that of MG. The level of carotenoids was similar between the two parental lines (Figure 2). In addition, the spotted part of SC had a higher chlorophyll content than the non-spotted part, while no difference was found in carotenoid contents between the spotted and non-spotted part of SC.

**FIGURE 3:**
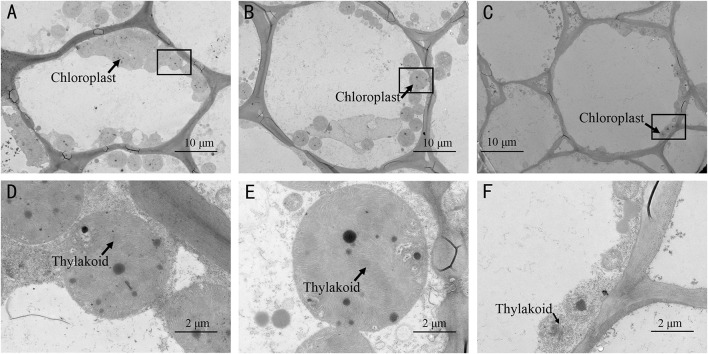
Transmission electron microscopy (TEM) observation of chloroplasts in the spotted peels of SC **(A,D)** and non-spotted peels of SC **(B,E)**, and MG **(C,F)**. **(D–F)** Magnified views of the boxed regions in **(A–C)**, respectively.

Furthermore, TEM observations showed that there were more chloroplasts in the rind cells of the spotted part of SC than that in MG or non-spotted part of SC (Figures 3A–C and Supplementary Table 3). There was no evident difference in the chloroplast numbers of the rind cells between the non-spotted part of SC and MG, while the chloroplast was much bigger in SC than that in MG (Figures 3D–F and Supplementary Table 3). Meanwhile, a much more tightly stacking lamella structure of chloroplast was obviously observed in the rind cells of SC than MG. Consequently, the mottled rind of melon was due to the abnormal development of chloroplast number and the chlorophyll accumulation during the fruit development. And the developmental state of thylakoid structure in chloroplasts might be the reason for the difference of immature rind color between the two parental lines.

### Genetic Analysis of Mottled Rind in Melon

To investigate the genetic inheritance of mottled rind in melon, reciprocal crosses between SC and MG were performed. The resulting F_1_ or F′_1_ plants exhibited the dark green and mottled rind (Figure 1C), similar to the parent, SC. In the F_2_ population, the segregation ratio was 9:7 (mottled/non-mottled ratio of 318:235, χ^2^ = 0.354) (Table 1). Among the BC_1_P_2_ population (171 individuals), 39 showed the mottled rind, and 132 showed the non-mottled rind, corresponding to a segregation ratio of 1:3 by the Chi-square test. These results indicated that two dominant and epistatic genes, namely, *CmMt1* and *CmMt2*, were involved in the genetics of the mottled rind pattern in SC-MG melon. It could be concluded that mottled rind pattern of melon fruit was controlled by two dominant complementary genes, comparing with non-mottled rind. Consequently, SC should contain two dominant alleles, while MG should contain two recessive alleles. Coincidentally, we identified a 9:3:4 segregation ratio for dark green mottled, dark green non-mottled, and light green non-mottled traits by analyzing the above F_2_ segregating population (Figures 1D–F and Table 2), indicating that the dominant allele of rind color gene is necessary for the development of the spots on the rind. Statistical evidence of deviation from the 1:1 expected segregation ratio was measured with a χ^2^ goodness-of-fit test in another BC_1_ segregating population derived from a cross between a dark green mottled rind line HPG and a dark green non-mottled rind line MR-5 (Supplementary Figure 1). And the segregation ratio fits a 1:1 ratio as determined by a Chi-square test (χ^2^ = 0.281, *p* = 0.596) (Supplementary Table 4), which further proved the abovementioned conclusions.

**TABLE 1:** Inheritance of the mottled rind in melon.

**Crosses**	**Generation**	**Mottled**	**Non-mottled**	**Expected ratio (M:N)**	**Chi-square**	***p* ^a^**
SC	P_1_	10	0			
MG	P_2_	0	10			
SC × MG	F_1_	20	0			
MG × SC	F′_1_	19	0			
(SC × MG) × MG	BC_1_P_2_	39	132	1:3	0.439	0.508
(SC × MG)⊗	F_2_	318	235	9:7	0.354	0.552

*M, mottled; N, non-mottled.*

*Phenotypic observations for the fruit rind were at 10–15 days after pollination (dap).*

*^a^Segregation ratios are statistically consistent with the expected ratios (χ^2^ test, *p* < 0.05).*

**TABLE 2:** Segregation ratio of rind patterns and immature rind color in the F_2_ populations.

**Crosses**	**Generation**	**Dark green**		**Light green**		**Expected ratio**	**Chi-square**	***p* ^a^**
		**Mottled**	**Non-mottled**	**Mottled**	**Non-mottled**			
(SC × MG)⊗	F_2_	318	102	0	133	9:3:4	0.381	0.944

*^*a*^Segregation ratios are statistically consistent with the expected ratios (χ^2^ test, *p* < 0.05).*

Previous studies have confirmed that *CmAPRR2* was the key gene controlling rind color in immature fruit rind, and the causative variants at the *CmAPRR2* alleles were found in the parental lines based on next-generation sequencing of genomic DNA (Oren et al., 2019; Xu et al., 2021). Therefore, genotyping was performed in the F_2_ segregating population with the developed KASP DNA marker (*CmAPRR2*^*SNP(G/T)*^). The results further validated that all the mottled individuals had one or two copies of *CmAPRR2* from the female parent (SC) (Supplementary Table 5) and that the genotyping results were consistent with the phenotype. In summary, those results indicated that the *CmAPRR2* regulating young fruit rind color in melon was the candidate epistatic gene (*CmMt1*) to the mottled rind. However, it is also possible that *CmAPRR2* was only closely linked to the target epistatic gene (*CmMt1*) and the results need to be further confirmed.

### Whole-Genome Resequencing-Bulked Segregant Analysis and Linkage Mapping of the *CmMt2* Locus

**FIGURE 4:**
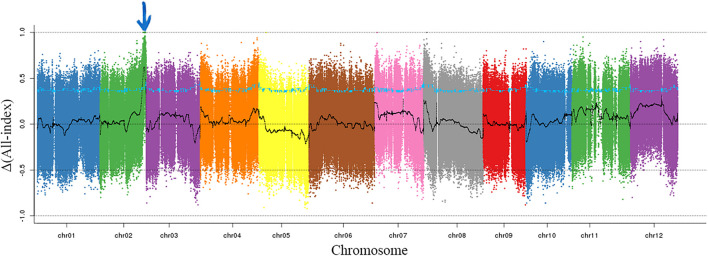
Δ(All-index) graphs from BSA-seq. The Δ(all-index) was calculated at a 1-Mb intervals with a 50-kb sliding window. One candidate region was identified on chromosome 2 indicating with the blue arrow.

To exclude the influence of epistatic gene, only the individuals with dark green rind were used to map the *CmMt2*. For BSA-seq, 20 mottled (M-pool) and 20 non-mottled (N-pool) F_2_ plants and two parental lines, such as SC and MG, were selected. In total, 13.6, 11.1, 16.9, and 14.6 GB of clean data were generated for M-pool, N-pool, SC, and MG, representing approximately 28-, 23-, 37-, and 30-fold genome coverage, respectively, based on the estimated genome size of 417 Mb. The clean reads of each sample were mapped to the reference genome of the DHL92 (version 4.0). After filtering, a total of 3,002,120 SNPs and 671,480 Indels, which were distributed merely on 12 chromosomes, were identified between the M-pool and the N-pool. To identify the causal region, the Δ(SNP-index) and Δ(Indel-index) of each position were calculated for sliding window analysis, and the results showed that the Δ(All-index) values were approximately 0.5 for most parts of the genome. A single 2.0-Mb genomic region on chromosome 2 was identified as the candidate region for mottled rind trait at a 95% significant level (Figure 4).

**FIGURE 5:**
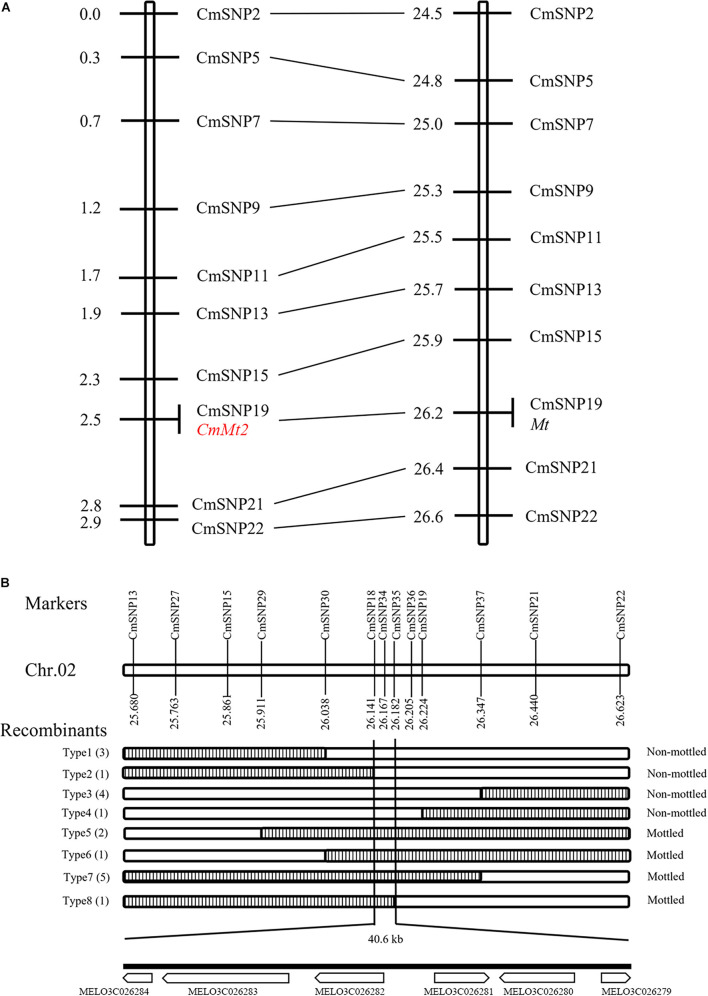
Initial and fine mapping of the *CmMt2* gene in melon. **(A)** Initial mapping of *CmMt2*. Genetic map of *CmMt2* is on the left, with cM as the unit. The corresponding physical map (right, unit: Mb) is also shown. **(B)** Fine mapping of *CmMt2*. The *CmMt2* gene was delimited to an interval between CmSNP18 and CmSNP35, with an estimated length of 40.6 kb, and six genes were annotated in this region based on the reference genome sequence. The genetic structure of each recombinant type is depicted as white for homozygous non-mottled rind and striped for heterozygous mottled alleles. The number of each recombinant type is indicated in bracket.

To precisely narrow down the candidate region, the resequencing data of SC and MG were analyzed to identify polymorphisms for the 2.0-Mb region between the two parental lines and develop KASP markers. Ten validated KASP markers were genotyped in 135 individuals with dark green rind, which was randomly selected from the F_2_ population. The initial linkage map was constructed using QTL IciMapping version 4.2, and the order of the markers in the map is consistent with that in the physical map. The results showed that the *CmMt2* locus was localized within the 0.5 cM regions, co-segregating with CmSNP19 (Figure 5A). All the dark green rind individuals of the F_2_ population were adopted to fine-map the *CmMt2* locus using flanking markers and eight newly developed markers. Consequently, the results delimited the *CmMt2* to a 40.6-kb region on chromosome 2 between the markers CmSNP18 and CmSNP35, each with one recombinant (type 2 and type 8) (Figure 5B).

### Candidate Gene Analysis

**FIGURE 6:**
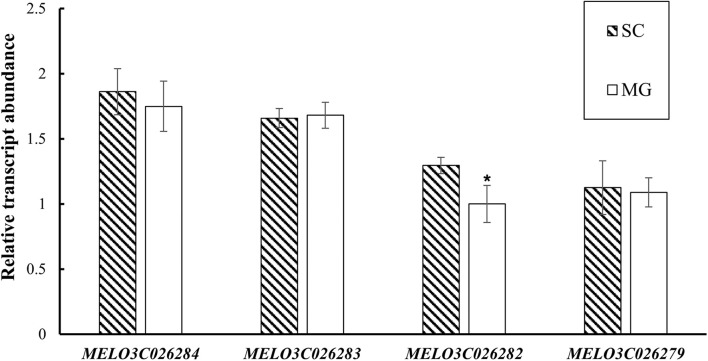
Expression evaluation of *MELO3C026284*, *MELO3C026283*, *MELO3C026282*, and *MELO3C026279* by qRT-PCR. The values are shown as mean ± SD of three biological and three technical replicates. *Significant differences (*t*-test, *p* < 0.05).

Based on the fine-mapping results of *CmMt2*, the DNA sequences in the abovementioned 40.6 kb interval were analyzed according to the “DHL92” melon genome (version 4.0). As a result, six predicted genes were identified in the mapping region (Supplementary Table 6). To pinpoint the candidate gene of *CmMt2*, the resequencing data of SC, MG, and the two BSA pools were analyzed by alignment. In total, 231 SNPs and 52 Indel were detected in the region corresponding to the reference genome. Among them, 266 mutation sites were in the intergenic region or intron region, and 17 were located in protein-coding gene regions, which were the sites of our concerns. For the 16 SNPs and 1 Indel, 5 SNPs caused a synonymous variant, while the other 12 variations caused a non-synonymous mutation between the two parents, indicating that these SNPs or Indel might be the causative sites for the mottled rind. These 12 variation sites were distributed in four genes, including *MELO3C026284*, *MELO3C026283*, *MELO3C026282*, and *MELO3C026279*. qRT-PCR was performed on 15 dap rinds of SC and MG plants to determine the candidate gene. The results revealed that the *MELO3C026282* was highly expressed in the mottled rind parent (SC) than in the non-mottled rind parent (MG), while the other three genes (i.e., *MELO3C026284*, *MELO3C026283*, and *MELO3C026279*) exhibited a similar transcript level between the two parents (Figure 6). Besides, *MELO3C026282* was predicted to encode a Sec14p-like phosphatidylinositol transfer family protein, homolog to *AtCPSFL1*, which is essential for chloroplast development (Pan et al., 2013). Taken together, our results indicated that *MELO3C026282* gene was the most likely candidate gene for *CmMt2*.

In the “DHL92” genome, *MELO3C026282* was 4,345 bp in length with 8 exons and encoded a protein of 261 amino acids. To characterize the sequence of the candidate gene in the parental lines, the coding sequences of *MELO3C026282* were amplified. The CDS of *MELO3C026282* in the parental lines was 786 bp in length. Sequence alignment between the parental lines revealed that there were five SNPs in the exon, including three base substitutions resulting in synonymous mutations and two base substitutions resulting in non-synonymous mutations. Conserved domain analysis in EMBL-EBI showed that the MELO3C026282 contained an N-terminal CRAL_TRIO domain and a C-terminal CRAL_TRIO domain with the chloroplast targeting sequences at the beginning. The two non-synonymous mutations leading amino acid substitution were in the N-terminal CRAL_TRIO domain, which may cause a loss of the conserved domain and ultimately lose the function of the CmMt2 protein.

## Discussion

Rind pattern is an important appearance quality of fruits, which is contributing to their commercial value (Liu G. et al., 2020). Therefore, the identification of genes or closely linked markers would be of great value for breeding, especially for fruits of horticultural crops (Sanseverino et al., 2015; Zhao et al., 2019). Previous studies showed that the spots on the melon fruit are a dominant trait controlled by a single gene (Liu et al., 2016; Lv et al., 2018; Pereira et al., 2018), whereas the mapping results are quite different and localized on chromosomes 2 and 4, respectively. We found that the mottled rind (spots on the rind) was controlled by two dominant genes (i.e., *CmMt1* and *CmMt2*) with epistatic effects and that two candidate genes were located close to previous reports. Due to the different parental materials, the conclusions are inconsistent. Here, we extended the genetic model of mottled rind and showed evidence of epistasis, which may explain the genetically diverse progenies in rind pattern crossing from the parents without spots on the rind (Li et al., 2019). Considering that the population was constructed with an inter-subspecific hybrid, our results reveal more genetic variation information about mottled rind in melon.

Coincidentally, the segregation ratio of rind traits showed that the rind color of immature fruit was an epistatic to the mottled rind, which is regulated by *CmAPRR2* (Oren et al., 2019; Xu et al., 2021). In addition, the genotyping results of *CmAPRR2* confirmed the results of phenotypic segregation. Previous reports indicated that the orthologous genes of *CmAPRR2* in cucumber (*Cucumis sativus* L.) (Liu et al., 2016), watermelon [*Citrullus lanatus* (Thunb.) Matsum. & Nakai] (Oren et al., 2019), pepper (*Capsicum annuum* L.), and tomato (*Solanum lycopersicum*) (Pan et al., 2013) have been proven to have similar functions controlling the chlorophyll accumulation on fruit rind. Compared with non-mottled rind, the number of chloroplasts and the chlorophyll content in the mottled rind were much higher. Thus, we suspected that *CmAPRR2* was the candidate gene for *CmMt1*. Based on the phenotype of the F_2_ individuals with a dark green rind, the *CmMt2* controlling mottled melon rind was mapped to an interval of 40.6 kb on chromosome 2 using BSA and KASP analysis. Further, according to the qRT-PCR results, the expression level of *MELO3C026282* in MG with non-mottled rind was significantly lower than that in SC with mottled rind. Therefore, *MELO3C026282* was the most likely candidate gene for *CmMt2*, which codes a Sec14p-like phosphatidylinositol transfer family protein playing a significant role in chloroplast development (Hertle et al., 2020). Studies in tomato and cucumber have confirmed that *APRR2* has the activity of transcription factors, closer with *GLK2-like* and *MYB* transcription factors family (Jiao et al., 2017). In monkeyflowers (*Mimulus*), an *R2R3-MYB* activator and an *R3-MYB* repressor produce spotted pigment patterning in flowers (Ding et al., 2020). Whether there is a similar system in melon needs to be further studied. And there were two SNPs in *MELO3C026282* coding sequences between the parental lines. The mechanism by which SNP affects the differential expression of *MELO3C026282* in the rind requires further investigation.

In addition, the mottled fruit is a common phenomenon in horticultural crops (Zhai et al., 2019), including pear (*Pyrus bretschneideri*), tomato, and apple (*Malus pumila*); however, the mechanism responsible for this uneven distribution of pigments remains largely unknown (Ding et al., 2020). In tomato, *TAGL1* was cloned, which regulated diversified chloroplast development and pigment accumulation (Hertle et al., 2020). Our study validated the mottled rind of melon was mainly caused by the difference in chloroplast development and chlorophyll accumulation. In summary, we assumed that *CmMt1* regulated the accumulation of chlorophyll and *CmMt2* might be involved in the development of chloroplast number, which cooperated to control the formation of mottled rind in melon.

## Conclusion

We reported that mottled rind in melon is controlled by two dominant genes with epistatic effects. The epistatic gene *CmMt1* was co-segregated with *CmAPRR2* controlling the pigment accumulation in immature melon fruit, which suggested that *CmAPRR2* was the candidate gene for *CmMt1*. Furthermore, the *CmMt2* locus was mapped between 24.53 and 26.57 Mb on chromosome 2 and fine-mapped in an interval of 40.6 kb with KASP analysis. The candidate gene *MELO3C026282* for *CmMt2* within the interval that is involved in the mottled rind formation was predicted through functional annotation, expression analysis, and sequence variation analyses. Our study provides insight into the molecular mechanism underlying rind appearance and reveals valuable information for marker-assisted selection breeding in melon.

## Data Availability Statement

The datasets presented in this study can be found in online repositories. The names of the repository/repositories and accession number(s) can be found below: https://www.ncbi.nlm.nih.gov/genbank/, PRJNA759737.

## Author Contributions

JS conceived and designed the study. XX performed the analyses. XN and YZ prepared the melon samples. JS, XX, and WS wrote the manuscript. All authors checked and approved the final version.

## Conflict of Interest

The authors declare that the research was conducted in the absence of any commercial or financial relationships that could be construed as a potential conflict of interest.

## Publisher’s Note

All claims expressed in this article are solely those of the authors and do not necessarily represent those of their affiliated organizations, or those of the publisher, the editors and the reviewers. Any product that may be evaluated in this article, or claim that may be made by its manufacturer, is not guaranteed or endorsed by the publisher.
