# Supplementary material for: Genetic Mapping and Identification of the Candidate Genes for Mottled Rind in Cucumis melo L
Source: Front Plant Sci. 2021 Nov 15;12:769989. doi: 10.3389/fpls.2021.769989 (PMC8634580; doi:10.3389/fpls.2021.769989)
Supplement: Supplementary file 1 [file Data_Sheet_1.docx]

Supplementary Material


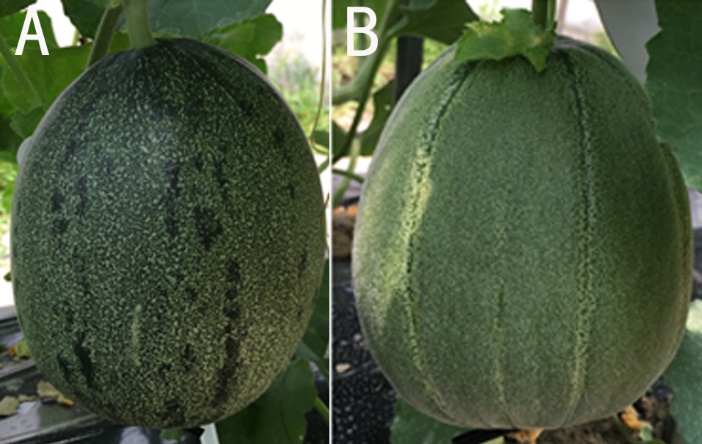


**Supplementary Figure 1.** Immature melon fruit of HPG (A, maternal parent, dark green and mottled rind) and MR-5 (B, paternal parent, dark green and non-mottled rind).
